# Quality of Life After Open Surgical *versus* Endovascular Repair of Abdominal Aortic Aneurysms

**DOI:** 10.21470/1678-9741-2017-0236

**Published:** 2018

**Authors:** Mustafa Akbulut, Eray Aksoy, İbrahim Kara, Davut Cekmecelioglu, Cengiz Koksal

**Affiliations:** 1 Department of Cardiovascular Surgery, Kartal Koşuyolu Research and Training Hospital, Kartal, Istanbul, Turkey.; 2 Department of Cardiovascular Surgery, Sakarya University Medical Faculty, Sakarya, Turkey.; 3 Department of Cardiovascular Surgery, Bezmialem Vakıf University Medical Faculty, Istanbul, Turkey.

**Keywords:** Quality of Life, Endovascular Procedures, Aortic Aneurysm, Abdominal

## Abstract

**Objective:**

This study aims to compare open surgical and endovascular aneurysm repair (EVAR) of abdominal aortic aneurysms in terms of their effects on quality of life, using Short Form-36 (SF-36).

**Methods:**

A total of 133 consecutive patients who underwent EVAR or open surgical repair for infra-renal abdominal aorta aneurysm between January 2009 and June 2014 were included in the study. Twenty-six (19.5%) patients died during follow-up and were excluded from the analysis. Overall, 107 patients, 39 (36.4%) in the open repair group, and 68 (63.6%) in the EVAR group, completed all follow-up visits and study assessments. Quality of life assessments using SF-36 were performed before surgery and at post-operative months 1, 6, and 12.

**Results:**

The mean duration of follow-up was 29.55±19.95 months. At one month, both physical and mental domains of the quality of life assessments favored EVAR, while the two surgical approaches did not differ significantly at or after six months postoperatively.

**Conclusion:**

Despite anatomical advantages and acceptable mid-phase mortality in patients with high- or medium-risk for open surgery, EVAR did not exhibit a quality of life superiority over open surgery in terms of physical function and patient comfort at or after postoperative six months.

**Table:** 

Abbreviations, acronyms & symbols	
AAA	= Abdominal aortic aneurysm
BP	= Somatic pain
EVAR	= Endovascular aneurysm repair
GH	= General health assessment
MH	= Mental health
PF	= Physical function
RE	= Emotional problems
RP	= Physical problems
SF	= Social function
SF-36	= Short Form-36
VT	= Energy/vitality
WHO	= World Health Organization

## INTRODUCTION

Endovascular aneurysm repair (EVAR) is a minimally invasive procedure that was originally developed to reduce the surgical stress levels in patients with a high risk for open surgical repair of abdominal aortic aneurysm (AAA) treatment. Comprehensive studies comparing success rates and outcomes in EVAR and open surgery revealed certain advantages of EVAR over open surgery including reduced blood loss, need for transfusions, need for mechanical ventilation, procedure duration, and intensive care as well as hospital stay^[^^1^^-^^4^^]^. A further benefit of EVAR was represented by the lower early mortality rate when compared with the open approach. Accordingly, the reported 30-day mortality rates in EVAR groups were 2.1% in EVAR-1 and 1.2% in DREAM trials, as compared to the respective figures of 6.2% and 4.6% for open surgery; however, this early mortality advantage of EVAR faded over time as a result of subsequent increase in mortality and complications requiring intervention^[^^1^^,^^5^^,^^6^^]^.

Although mortality and morbidity were the key criteria for evaluating the technical success rates of these procedures, health is a "state of complete physical, mental, and social well-being, and not merely the absence of disease" as defined by the World Health Organization (WHO). Therefore, the therapeutic approaches should not only aim at prolonging life, but also improving the quality of life. When choosing among therapeutic alternatives, health quality and patient comfort associated with a certain procedure should also be given a consideration in addition to risk assessments. Thus, the present study compared these two surgical repair methods for abdominal aortic aneurysms with respect to their effects on the quality of life.

## METHODS

The target sample population for this single-center study included 133 consecutive patients who underwent aortic aneurysm repair due to infra-renal abdominal aortic aneurysm between January 2009 and June 2014. There were 26 cases of mortality during the follow-up period. Therefore, a total of 107 patients who completed all follow-up assessments were included in the study analyses. Of these patients, 39 (36.4%) and 68 (63.6%) were in the surgical repair and EVAR groups, respectively. The mean duration of follow up was 29.55±19.95 months (range: 1-78 months). Prior to study procedures, study protocol was approved by the institutional ethics board.

Turkish version of Short Form 36 (SF-36) was used for the evaluation of the quality of life. Validity and reliability of Turkish version has been previously shown^[^^7^^]^. In summary, SF-36 is a 36-item, patient-reported outcome measure divided into 8 subscales in 2 domains, *i.e.* physical and mental health. The physical health domain evaluates physical function (PF), role constraints due to physical problems (RP), somatic pain (BP) and general health assessment (GH), while the mental health domain assesses social function (SF), role constraints due to emotional problems (RE), mental health (MH), energy/vitality (VT). The scale assesses the health status within the past four-week period.

All participants completed SF-36 preoperatively as well as at postoperative months 1, 6, and 12. For the purpose of the study analyses, scores were evaluated using the coefficients calculated for Turkish standards.

### Statistical Analysis

IBM SPSS Statistics 22 (IBM SPSS, Turkey) software was used for statistical analyses. Shapiro Wilks test was used to test the normality of the data. In addition to descriptive statistics (mean, standard deviation, frequency), Student's t-test was used for comparing quantitative data with normal distribution between the two groups, while Mann-Whitney U test was used for the comparison of data without normal distribution. Within group, comparisons for parameters without normal distribution were performed using Wilcoxon Sign Test. For the comparison of qualitative data, Chi-Square test, Fisher's Exact test, and Yate's Continuity Correction were used. Significance was set at a *P* level of less than 0.05.

## RESULTS

**Fig. 1:**
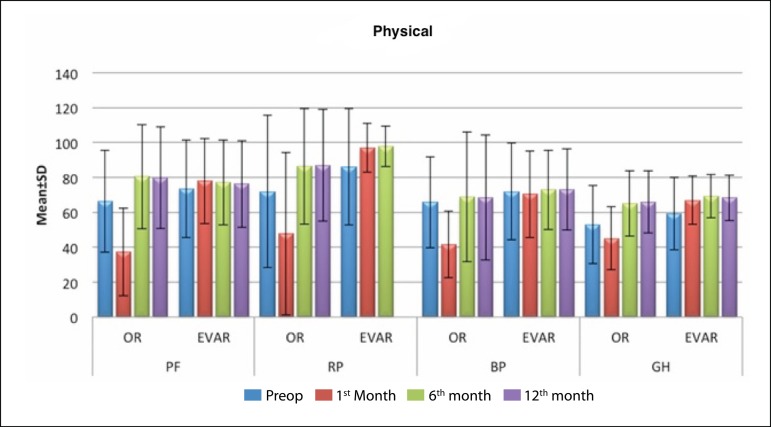
Changes in physical domain scores over time. BP=body pain; EVAR=endovascular aneurysm repair; GH=general health; OR=open repair; PF=physical function; RP=role constraints due to physical problems

**Fig. 2:**
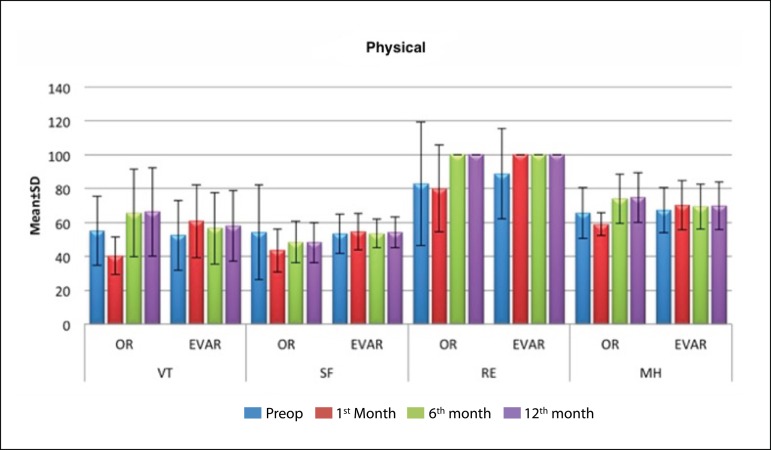
Changes in mental domain scores over time. EVAR=endovascular aneurysm repair; OR=open repair; MH=mental health; RE=role constraints due to emotional problems; VT=energy/vitality

Clinical characteristics of the patients in study groups are shown in Table 1. The two groups, *i.e*. endovascular *vs.* open surgical repair groups, were comparable in terms of demographic and clinical characteristics (*P*>0.05), except for more frequent low ejection fraction (<40%) in the EVAR group (P=0.013). The mortality rates were 29.09% (n=16) and 12.82% (n=10) for open surgical and EVAR groups, respectively. The changes in composite physical and mental scores over time in study groups are shown in Figure 1, 2, and Table 2. Preoperatively, SF-36 scores in the two study groups were similar (*P*>0.05).

**Table 1:** Comparison of the groups with respect to preoperative parameters.

		Open repair	EVAR	*P*
Age (year) mean±SD		67.22±8.92	67±9.12	0.891
Gender n,%	Female	6 (10.9%)	10 (12.8%)	0.950
	Male	49 (89.1%)	68 (87.2%)	
Family history n,%		35 (63.6%)	45 (57.7%)	0.610
Smoking n,%		44 (80%)	65 (83.3%)	0.792
BMI >29 n,%		30 (54.5%)	43 (55.1%)	1.000
HL (LDL>100) n,%		34 (61.8%)	56 (71.8%)	0.306
DM n,%		17 (30.9%)	24 (30.8%)	1.000
HT n,%		43 (78.2%)	69 (88.5%)	0.174
CRF n,%		13 (23.6%)	24 (30.8%)	0.479
COPD (FEV<1) n,%		24 (43.6%)	35 (44.9%)	1.000
PAD n,%		5 (9.1%)	8 (10.3%)	1.000
CAD n,%		29 (52.7%)	49 (62.8%)	0.244
EF <40 n,%		4 (7.3%)	20 (25.6%)	0.013
Carotid artery disease n,%		10 (18.2%)	16 (20.5%)	0.911
CVA		3 (5.5%)	4 (5.1%)	1.000
Aneurysm diameter	>5,5-7	30 (54.5%)	44 (56.4%)	0.831
	>7	25 (45.5%)	34 (43.6%)	

BMI=body mass index; CABG=coronary artery bypass grafting; CAD=coronary artery disease; COPD=chronic obstructive pulmonary disease; CRH=chronic renal failure; CVA=cerebrovascular accident; DM=diabetes mellitus; EF=ejection fraction; HL=hyperlipidemia; HT=hypertension; PAD=peripheral arterial disease

**Table 2:** Changes in SF-36 scores over time.

		Preoperative	1^st^ month	6^th^ month	12^th^ month
		Mean±SD (median)	Mean±SD (median)	Mean±SD (median)	Mean±SD (median)
PF	Open repair	66.36±29.16 (80)	37.27±25.07 (35)	80.48±29.89 (100)	79.85±29.09 (90)
	EVAR	73.56±27.98 (90)	77.97±24.43 (95)	77.07±24.26 (92.5)	76.25±24.86 (92.5)
	^1^ *P*	0.112	0.001**	0.314	0.410
RP	Open repair	71.97±43.64 (100)	47.73±46.5 (25)	86.36±33.13 (100)	87.04±32.05 (100)
	EVAR	86.02±33.24 (100)	97.03±13.99 (100)	97.83±11.58 (100)	97.83±11.58 (100)
	^1^ *P*	0.128	0.001**	0.040*	0.072
BP	Open repair	65.82±26.05 (72)	41.7±19.14 (42)	68.85±37.13 (100)	68.52±35.78 (74)
	EVAR	71.98±27.75 (80)	70.42±24.85 (74)	72.87±22.65 (74)	73.18±23.24 (74)
	^1^ *P*	0.318	0.001**	0.652	0.903
GH	Open repair	53.03±22.41 (57)	45.03±18.07 (35)	64.97±18.59 (72)	66±17.8 (72)
	EVAR	59.34±20.77 (62)	66.81±13.84 (67)	69.17±12.38 (72)	68.29±13.04 (72)
	^1^ *P*	0.192	0.001**	0.648	0.965
VT	Open repair	55±20.31 (55)	40.3±11.04 (45)	65.61±25.76 (75)	66.3±26.11 (75)
	EVAR	52.46±20.67 (45)	60.76±21.58 (60)	56.63±21.16 (47.5)	58.04±20.83 (50)
	^1^ *P*	0.422	0.001**	0.072	0.133
SF	Open repair	54.17±27.89 (62.5)	43.45±12.84 (37.5)	48.48±12.4 (50)	48.15±11.86 (50)
	EVAR	53.38±11.72 (50)	54.64±10.77 (50)	53.48±8.46 (50)	54.13±9.01 (50)
	^1^ *P*	0.321	0.001**	0.217	0.188
RE	Open repair	82.83±36.44 (100)	80.1±25.61 (100)	100±0 (100)	100±0 (100)
	EVAR	88.89±26.7 (100)	100±0 (100)	100±0 (100)	100±0 (100)
	^1^ *P*	0.509	0.001**	1	1
MH	Open repair	65.45±15.03 (64)	59.03±6.93 (60)	74.06±14.7 (72)	74.67±14.63 (72)
	EVAR	67.12±13.25 (68)	70.1±14.52 (68)	69.3±13.25 (64)	69.86±14.05 (64)
	^1^ *P*	0.733	0.001**	0.091	0.173

BP=body pain; EVAR=endovascular aneurysm repair; GH=general health; PF=physical function; MH=mental health; RE=role constraints due to emotional problems; RP=role constraints due to physical problems; SF=social function; VT=energy/vitality;

** P<0.05

At postoperative month 1, patients in EVAR group had significantly higher (*i.e.* better) SF-36 scores as compared to those in the open surgery group in all 8 sub-score domains (*P*<0.01). However, this difference in mental and physical health domain scores between the two groups disappeared at months 6 and 12 (*P*>0.05).

## DISCUSSION

Previous trials comparing open surgical repair *vs.* endovascular stent grafting for the management of abdominal aneurysms in terms of medium and long-term mortality and morbidity rates have provided comparable outcomes for the two approaches^[^^1^^,^^5^^,^^6^^]^. On the other hand, EVAR offered certain morbidity advantages such as reduced need for blood transfusions as well as shortened intensive care and hospital stay^[^^8^^-^^10^^]^.

EVAR is preferred in high risk or elderly patients, or in those with comorbid conditions based on the minimally invasive nature of the procedure, especially when anatomically feasible. The success of EVAR or open surgical repair for the treatment of abdominal aortic aneurysms has been evaluated through extensive clinical research focusing mainly on mortality and morbidity rates^[^^1^^,^^5^^,^^6^^]^. However, improved life expectancy may represent only one dimension of a treatment effect, which also has an impact on the psychological well-being as well as the patient comfort. Therefore, in this study, two different surgical approaches for the management of abdominal aortic aneurysms were compared with regard to their effects on the quality of life using SF-36, in order to assist the decision-making process prior to surgery in such patients.

SF-36 has been validated as a reliable tool for assessing well-being and health perception from patients' viewpoint. Specifically, vascular surgery societies also have been endorsing the use of SF-36 for follow-up life quality assessments of patients undergoing vascular surgery^[^^11^^]^.

In a study by Malina et al.^[^^12^^]^, despite lower physical health scores in the first 30 days after surgery among patients undergoing open surgery as compared to those undergoing EVAR, this difference favoring EVAR disappeared at postoperative month 3. In our study, there was a marked reduction in physical and mental health scores during the first postoperative month among open repair patients, while no statistically significant changes in these scores occurred in EVAR group. As earlier pointed out by Chetter et al.^[^^13^^]^, energy/vitality represents the single most important determinant of patients' mood. This suggests that the subjective energy level of the patient may be a primary factor driving mental health differences in different surgery groups. In the study by Malina et al.^[^^12^^]^, mental health scores among the patients showed an increase above the baseline at 3 months after surgery, coinciding with the completion of recovery phase and removal of the life-threatening situation from patients' point of view. It therefore appears that reduced anxiety correlates with improved quality of life.

Lloyd et al.^[^^14^^]^ examining these two surgical methods found that life quality scores returned to baseline at six months in both groups, and equaled at 12 months. In another study, although physical health scores were lower than baseline during the first postoperative month in EVAR patients, both physical and mental scores returned to baseline levels at postoperative months 3 and 12^[^^6^^]^. Furthermore, in some previous studies quality of life in patients undergoing open surgery was worse compared to the normal population during the early postoperative period, and EVAR was also reported to result in worse outcomes in the longer term^[^^15^^,^^16^^]^. In our study, the two groups did not significantly differ with respect to quality of life scores at or after 6 months postoperatively.

Based on its minimal invasive nature, endovascular stent graft repair may be expected to positively affect the quality of life in patients. However, the need for close monitoring of the patient for endo-leaks, graft failure, and continued expansion of the aneurysmal sac may have an adverse impact on the quality of life, particularly when one considers the potential requirement for re-surgery with open or endovascular approaches as a result of complications^[^^17^^]^. Therefore, the weight of evidence suggests that the initial quality of life advantage of EVAR fades over time^[^^18^^-^^20^^]^, consistent with our observations showing no difference in terms of physical and mental health scores between the two arms at or after 6 months postoperatively.

Although our study is prospective in nature, one of its potential limitations is the absence of randomization.

## CONCLUSION

The results of our study show a significant positive effect of EVAR on both physical and mental aspects of health as compared to open aneurysm repair during the early postoperative period. However, this early advantage disappears with longer term follow up and the two methods become indistinguishable with regard to life quality effects. Therefore, endovascular repair may represent a better surgical option in elective cases with high comorbidity.

**Table:** 

Authors' roles & responsibilities	
MA	Substantial contributions to the conception or design of the work; or the acquisition, analysis, or interpretation of data for the work; final approval of the version to be published
EA	Drafting the work or revising it critically for important intellectual content; final approval of the version to be published
IK	Agreement to be accountable for all aspects of the work in ensuring that questions related to the accuracy or integrity of any part of the work are appropriately investigated and resolved; final approval of the version to be published
DC	Drafting the work or revising it critically for important intellectual content; final approval of the version to be published
CK	Final approval of the version to be published
